# Transgastric decompression using a newly developed nasojejunal tube in a postgastrostomy patient with adhesive small bowel obstruction

**DOI:** 10.1002/ccr3.1327

**Published:** 2018-01-13

**Authors:** Haruna Nakamura, Toh Yoon Ezekiel Wong

**Affiliations:** ^1^ Department of Internal Medicine (Gastroenterology) Hiroshima Kyoritsu Hospital Hiroshima City Japan

**Keywords:** Nasojejunal tube, small bowel obstruction, transgastric decompression

## Abstract

For postgastrostomy patients suffering from adhesive small bowel obstruction, transgastric decompression with a multiluminal nasojejunal tube is an effective and more comfortable alternative to conventional nasal tubing using an ileus tube.

## Clinical Image

**Figure 1:**
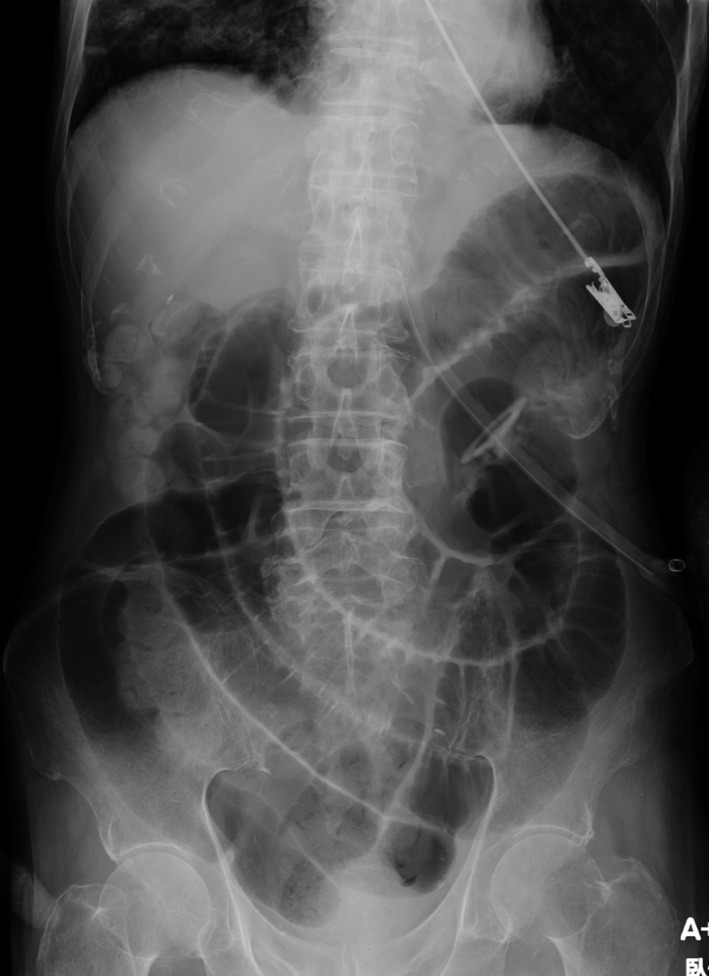
Abdominal radiograph revealing marked dilation of the small bowel due to adhesive ileus.

An 82‐year‐old bedridden patient being fed via a gastrostomy tube was admitted for severe aspiration pneumonia after persistent emesis. About 15 years ago, he underwent laparotomy for colorectal cancer and abdominal radiograph during admission showed marked dilation of the small bowel (Fig. 1), compatible with the diagnosis of adhesive small bowel ileus.

### Is nasal tubing the only option?

**Figure 2:**
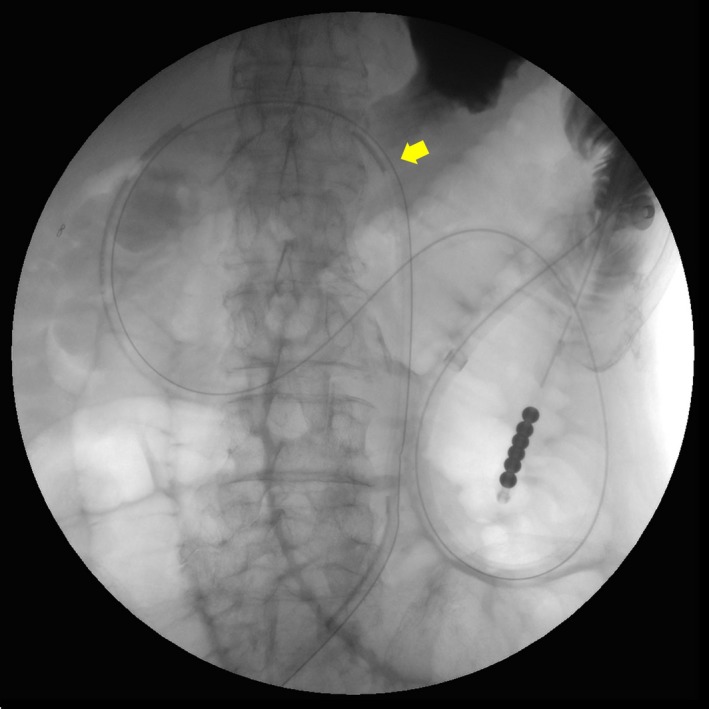
A newly developed nasojejunal tube was inserted via the gastrostomy site (yellow arrow) into the jejunum on day 2.

**Figure 3:**
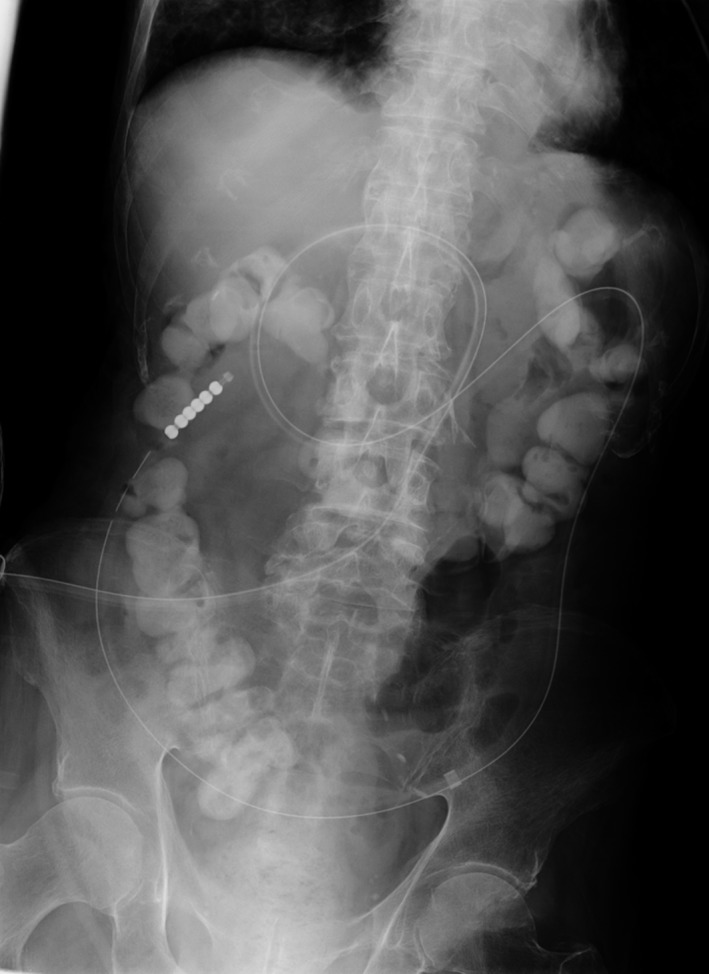
Successful decompression confirmed by abdominal radiograph on day 4.

Drainage with the existing gastrostomy tube was ineffective. However, instead of using a conventional transnasal ileus tube, a less expensive newly developed nasojejunal tube 1 was inserted via the gastrostomy site into the jejunum on day 2 (Fig. 2). Transgastric decompression was successful with the tube, as confirmed by an abdominal radiograph on day 4 (Fig. 3), and the patient eventually resumed enteral nutrition via gastrostomy. Management with this method, which was first reported by Shinoda et al. 2, not only avoided the unnecessary discomfort from nasal tubing but also enabled early jejunal feeding after resolution of the obstruction.

## Authorship

HN: prepared the manuscript. EWTY: supervised the preparation of the manuscript and management of the patient.

## Conflict of Interest

None to declare.
